# TIMP-2/IGFBP7 predicts acute kidney injury in out-of-hospital cardiac arrest survivors

**DOI:** 10.1186/s13054-018-2042-9

**Published:** 2018-05-12

**Authors:** Christoph Adler, Tobias Heller, Felix Schregel, Henning Hagmann, Martin Hellmich, Joana Adler, Hannes Reuter

**Affiliations:** 1 Department of Internal Medicine III, Division of Cardiology, Pneumology, Angiology and Intensive Care, University of Cologne, 50924 Cologne, Germany; 2 Department of Internal Medicine II, Division of Nephrology, Rheumatology, Diabetology and General Interne Medicine, University of Cologne, Cologne, Germany; 3 Institute of Medical Statistics, Informatics and Epidemiology, University of Cologne, Cologne, Germany; 4 Department of Internal Medicine and Cardiology, Ev. Klinikum Cologne-Weyertal, Cologne, Germany

**Keywords:** Acute kidney injury, Cardiac arrest, Shock, Target temperature management, Biomarker

## Abstract

**Background:**

Acute kidney injury (AKI) is a common complication after cardiopulmonary resuscitation (CPR) and predicts in-hospital mortality. To which extent post-resuscitation disease or the initial event of cardiac arrest and the duration of insufficient cardiac output triggers AKI is challenging to discriminate. Knowledge on molecular mediators of AKI is scarce. Early identification of patients at high risk of AKI is hampered by the low sensitivity of the established tests in clinical routine practice. The present study aimed to determine the diagnostic utility of the novel urine biomarkers tissue inhibitor of metalloproteinases-2 (TIMP-2) and insulin-like growth factor-binding protein 7 (IGFBP7) for the early recognition of AKI in patients with non-traumatic shock.

**Methods:**

The performance of [TIMP-2]**·**[IGFBP7] was prospectively analysed in 48 patients with shock following out-of-hospital cardiac arrest (OHCA). All patients were treated with target temperature management (TTM) for 24 h. Urinary [TIMP-2]**·**[IGFBP7] samples were collected at 3 and 24 h after determination of OHCA.

**Results:**

Patients (n = 31 (65%)) developed AKI after an average of 26 ± 12 h. Patients who developed AKI had significantly higher [TIMP-2]·[IGFBP7] compared to individuals that did not develop AKI (1.52 ± 0.13 vs. 0.13 ± 0.14; *p* < 0.05) as early as 3 h after determination of OHCA,. For urine [TIMP-2]*[IGFBP7], the area under the curve (AUC) for the development of AKI was 0.97 (CI 0.90–1.00) at 3 h after OHCA. The optimal [TIMP-2]·[IGFBP7] cut-off value for the prediction of AKI was 0.24. The sensitivity was 96.8% and specificity was 94.1%.

**Conclusions:**

Urinary [TIMP-2]•[IGFBP7] reliably predicts AKI in high-risk patients only 3 h after determination of OHCA with a cut-off at 0.24. This novel test may help to identify patients at high risk of AKI to enrol into clinical studies to further elucidate the pathophysiology of AKI and devise targeted interventions in the future.

**Electronic supplementary material:**

The online version of this article (10.1186/s13054-018-2042-9) contains supplementary material, which is available to authorized users.

## Background

Acute kidney injury (AKI) has been identified as an independent risk factor for morbidity and mortality in patients after out-of-hospital cardiac arrest (OHCA). Mechanisms that contribute to the development of AKI are not yet fully understood. There is some evidence that AKI is influenced by post-resuscitation disease and may not just be a consequence of cardiac arrest and time without spontaneous circulation [1]. The post-resuscitation disease is multifactorial and in addition to myocardial dysfunction it may include systemic inflammatory response syndrome (SIRS) mediated by cytokine release and expression of inducible nitric oxide synthase (NOS) among others [2, 3]. Excessive NOS results in high levels of nitric oxide, which may lead to inappropriate systemic vasodilatation, progressive systemic and coronary hypoperfusion, and myocardial depression [2, 4]. Furthermore, patients are likely to undergo diagnostic or therapeutic procedures like percutaneous coronary intervention (PCI) or computed tomography angiography (CTA) after OHCA, thus imposing an additional risk of AKI associated with the exposure to contrast agents.

Tujjar et al. reported a 43% incidence of AKI (defined as a daily urine output < 0.5 ml/kg/h and/or an increase in serum creatinine ≥ 0.3 mg/dl from admission value within 48 h or a 1.5-fold increase from baseline level) in patients with cardiac arrest [5]. Other studies observed an incidence of AKI after cardiac arrest ranging widely from 10 to 50% [6–10]. However, current diagnostic criteria, such as serum creatinine levels or oliguria have limited predictive value and appear late in the course of AKI. Novel diagnostic tools and biomarkers are urgently needed to allow for timely identification and stratification of patients at risk. Investigating this population for the mechanisms of AKI will aid the identification of drug targets and monitoring of their effectiveness in clinical trials.

Recently Kashani et al. reported the validation of two novel urinary biomarkers for AKI in critically ill patients [11]. Tissue inhibitor of metalloproteinases-2 (TIMP-2) and insulin-like growth factor-binding protein 7 (IGFBP7) are both G1 cell cycle arrest biomarkers, which are elevated in cases of stress or damage of the renal tubular cells. Combined urinary TIMP-2 and IGFBP7 have been shown to perform better than other known biomarkers for risk stratification in AKI in a heterogeneous group of critically ill patients. Risk of death, dialysis, or persistent renal dysfunction was significantly higher for [TIMP-2]·[IGFBP7] above 0.30 [11]. Bihorac and co-workers assessed the predictive cut-off values of [TIMP-2]·[IGFBP7] in a multi-centre trial of more than 400 critically ill patients. In this general ICU setting, [TIMP-2]·[IGFBP7] > 0.30 indicated a sevenfold increase in the risk of AKI. The area under the receiver operating characteristic (ROC) curve was 0.82 for the development of AKI [12]. Similar results were found in a study of patients who had undergone cardiac surgery for cardiopulmonary bypass. For urine [TIMP-2]*[IGFBP7], the area under the ROC curve was 0.81 at 4 h after surgery [13].

The present study aimed to determine the diagnostic utility of the novel urine biomarkers [TIMP-2]**·**[IGFBP7] for the early recognition of AKI in patients with non-traumatic shock following OHCA.

## Methods

Patients with non-traumatic out-of-hospital cardiac arrest (OHCA) admitted to the cardiac ICU of the University Hospital of Cologne between May 2014 and January 2016 were consecutively enrolled. Adults (≥ 18 years) who met the inclusion criteria (OHCA, return of spontaneous circulation (ROSC), Glasgow Coma Score (GCS) ≤ 8 and non-traumatic shock) were included. Pregnant patients and patients with chronic renal replacement therapy were excluded from the study. Patients who died within 24 h after admission or received extracorporeal membrane oxygenation (ECMO) therapy were also excluded from further analysis.

AKI was classified according to the Kidney Disease Improving Global Outcomes (KDIGO) guidelines during the patient’s hospital stay [14]. On this basis, patients with an increase in serum creatinine or reduced urine secretion were categorized into AKI stages 1–3. For statistical analyses, patients with AKI stages 1–3 were defined as the “AKI group”. Renal function was continuously monitored by determination of urinary secretion and serum creatinine. Patients with a previous medical history of any chronic kidney disease (CKD) and patients presenting with elevated serum creatinine (> 1.2 mg/dl) were defined as patients with “advanced renal disease”. The individual reference value for serum creatinine was obtained whenever possible from the family physician. Otherwise, the lowest serum creatinine value from the hospital stay was used [15]. Cardiac arrest was defined as apnoea or agonal respiration in a comatose patient receiving cardiopulmonary resuscitation (CPR). Time to ROSC was calculated from the time of determination of collapse to ROSC.

All patients with myocardial ischaemia as the suspected cause of cardiac arrest were transferred to the catheter laboratory for coronary angiography and revascularization when indicated prior to admission to the ICU. The diagnosis of myocardial infarction was adjudicated after angiographic confirmation of coronary artery occlusion. Patients with septic shock underwent further diagnostic evaluation, with x-ray, ultrasound or computer tomography applied for identification of the septic focus. Criteria for septic and cardiogenic shock were adopted from the current sepsis guidelines and the modified version from the SHOCK-trial [16, 17]. Patients were treated with target treatment management (TTM) for 24 h according to our standard operating procedure (SOP). Peripheral cooling with ice packs to the femoral and/or neck area was initiated in patients with OHCA by the first responding emergency medical service. The controlled cooling to a target temperature of 32–34 °C was continued in the ICU using an endovascular cooling device (Thermogard XP® catheter, Zoll Medical Corp., Chelmsford, MA, USA) and maintained for 24 h. TTM was terminated by rewarming through the same endovascular device at a controlled rate of 0.3 °C/h until the physiologic body temperature of 36.5 °C was reached.

All patients received standard intensive care treatment according to current recommendations. Patients were intubated and mechanically ventilated and received midazolam for sedation and sufentanil for analgesia aiming at a Richmond agitation and sedation score (RASS) of − 4 until rewarming and haemodynamic stabilisation. Medical treatment with vasopressors (norepinephrine or in individual cases also epinephrine) and inotropes (dobutamine) was used when indicated, to achieve a target mean arterial blood pressure ≥ 65 mmHg. Heart rate, pulse oximetry, invasive blood pressure and central venous pressure were monitored routinely. Core temperature was continuously registered in the bladder through a thermal sensor at the tip of a transurethral urinary catheter. In all patients with shock continuous ICU monitoring was complemented by transpulmonary thermodilution and arterial pulse contour analysis by the use of the VolumeView® set (Edwards Lifesciences, CA, USA). The VolumeView® system was routinely calibrated by thermodilution with 20 ml ice cold saline every 6 h. A minimum of three consecutive measurements was obtained and a difference of < 10% between the measurements was accepted for data collection. Crystalloid solution was used for fluid therapy. The individual body weight was obtained whenever possible from the family members, otherwise body weight was estimated.

Neurological outcome was registered in all patients one month after CPR based on the Glasgow-Pittsburgh cerebral performance categories (CPC) of the Utstein recommendations [18]. For analyses, neurologic outcome was dichotomized into favourable (CPC 1–2) and poor (CPC 3–5).

### Measurement of blood samples and urine biomarker assays

Urine samples were collected at predetermined time points: 3 h and 24 h after determination of OHCA. [TIMP-2]**·**[IGFBP7] were measured directly with the NephroCheck™ point-of-care test (Astute Medical Inc., San Diego, CA, USA). This bedside test uses a fluorescent immunoassay to measure IGFBP7 and TIMP-2 biomarkers per 100 μl urine. The test takes 20 min and multiplies the concentration of both biomarkers. The product is divided by 1000 to report a single numerical test result with a unit of (ng/ml)2/1000. This unit was used for all [TIMP-2]·[IGFBP7] values throughout the present report. Samples for serum creatinine, urea and cystatin C were collected in standard serum tubes (Sarstedt AG, Nuembrecht, Germany) and were processed routinely by the Institute for Clinical Chemistry of the University Hospital of Cologne.

### Study endpoint

The objective was the evaluation of the diagnostic utility of the novel urine biomarkers [TIMP-2]·[IGFBP7] for the early recognition of AKI in patients with non-traumatic shock following out-of-hospital cardiac arrest (OHCA).

### Statistical analysis

Distributions of categorical variables were summarized by count and percentage and of continuous variables by mean ± standard deviation (SD), contingent on distributional characteristics. Categorical variables were compared between groups by Fisher’s exact test and continuous data by Student’s *t* test. ROC analysis was used to determine the diagnostic accuracy of biomarkers for discrimination between AKI and non-AKI. Optimal cut-point selection was based on maximization of the Youden index (i.e. sensitivity + specificity −1). The added value of assessing [TIMP-2]·[IGFBP7] was evaluated in patients with AKI in a multivariable modelling approach. Penalized maximum logistic regression (Firth regression) was used to overcome the problem of “separation”. The difference between two values was defined as the absolute delta (Δ) value. Statistical analysis was performed using SPSS Statistics (version 24; IBM Corp., Armonk, NY, USA). A *p* value <0.05 was considered statistically significant.

## Results

### Baseline characteristics and study endpoint

**Fig. 1:**
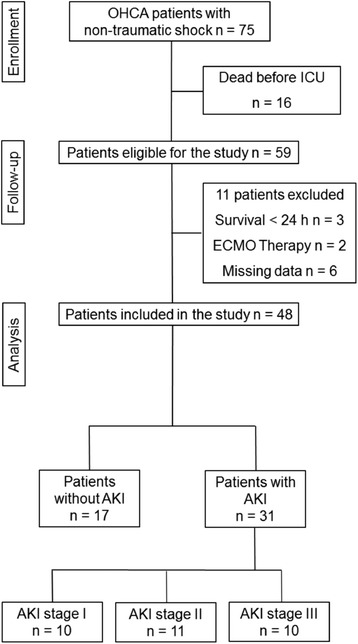
Patient disposition. OHCA, out-of-hospital cardiac arrest; AKI, acute kidney injury; ECMO, Extracorporeal membrane oxygenation

Seventy-five consecutive patients with non-traumatic shock following OHCA were admitted to the hospital during the study period. Of these, 16 patients died due to refractory shock before ICU admission. Eleven patients were excluded from the analysis due to different reasons (Fig. 1). Forty-eight patients with a mean age of 63 ± 11 years were analysed in the study: 87.5% of the examined patients presented with cardiogenic shock. Myocardial infarction was the most common cause of cardiac arrest (CA) (67% of patients) followed by primary arrhythmia (21% of patients). In almost 12.5% of patients, OHCA resulted from septic shock (five patients with pulmonary focus and one patient with serious skin infection). There were no differences between the two groups in age, medical history or first recorded rhythm. AKI developed more frequently in patients with unobserved CA. In total the amount of epinephrine applied during resuscitation was significantly greater in patients who developed AKI (4.5 ± 4.1 mg vs. 1.5 ± 2.0 mg; *p* < 0.05). In addition, longer CPR, measured as duration from determination of collapse to ROSC, resulted in an increased risk of AKI (25 ± 17 min vs.14 ± 5 min; *p* < 0.05). The amount of contrast agent applied during coronary angiography and coronary intervention was not significantly different between the AKI group and the group that did not develop AKI (163 ± 72 ml vs. 151 ± 61 ml, *p* = 0.61). Clinical characteristics of patients included in the present investigation are summarized in Table 1**.**

**Table 1:** Clinical characteristics of the patients included in the investigation

Characteristic	Study population (n = 48)	Patients with AKI (n = 31)	Patients without AKI (n = 17)	*P* value
Age (years)	63 ± 11	63 ± 12	62 ± 9	0.84
Female – *n*/total *n* (%)	4/48 (8)	2/31 (7)	2/17 (12)	0.61
Medical history				
Diabetes mellitus – *n*/total *n* (%)	8/48 (17)	5/31 (16)	3/17 (18)	1
Arterial hypertension – *n*/total *n* (%)	25/48 (52)	15/31 (48)	10/17 (59)	0.56
Coronary heart disease – *n*/total *n* (%)	42/48 (88)	28/31 (90)	14/17 (82)	0.68
Advanced renal disease – *n*/total *n* (%)	11/48 (23)	7/31 (23)	4/17 (22)	0.42
Cause of cardiac arrest				
Cardiogenic shock	42/48 (87.5)	27/31 (87)	15/17 (88)	1
Myocardial infarction – *n*/total *n* (%)	32/48 (67)	23/31 (74)	9/17 (59)	0.20
- Primary arrhythmia – *n*/total *n* (%)	10/48 (21)	4/31 (13)	6/17 (35)	0.14
Septic shock	6/48 (12.5)	4/31 (16)	2/17 (12)	1
- Pulmonary focus – *n*/total *n* (%)	5/48 (10)	3/31 (10)	2/17 (12)	1
- Skin infection – *n*/total *n* (%)	1/48 (2)	1/31 (3)	0/17 (0)	1
Witnessed cardiac arrest – *n*/total *n* (%)	33/48 (69)	18/31 (58)	15/17 (88)	<0.05
Basic life support provided by bystander – *n*/total *n* (%)	18/48 (38)	13/31 (42)	5/17 (29)	0.50
Ventricular fibrillation or pulseless ventricular tachycardia – *n*/total *n* (%)	41/48 (85)	26/31 (84)	15/17 (88)	1
Number of defibrillations/shocks	3.3 ± 2.5	3.6 ± 2.7	2.8 ± 1.8	0.36
Dose of epinephrine during CPR (mg)	3.3 ± 3.8	4.5 ± 4.1	1.5 ± 2.0	<0.05
Time from determined collapse to ROSC (min)	23 ± 19	25 ± 17	14 ± 5	<0.05
Outcome				
Period of ICU hospitalization (days)	17 ± 12	19 ± 9	16 ± 19	0.42
Ventilation time (days)	10 ± 9	9 ± 6	12 ± 9	0.50
Hospital mortality – *n*/total *n* (%)	14/48 (29)	13/31 (42)	1/17 (6)	<0.01
Favourable neurological outcome – *n*/total *n* (%)	29/48 (60)	15/31 (48)	14/17 (82)	0.03

*AKI* acute kidney injury, *CPR* cardiopulmonary resuscitation, *ROSC* return of spontaneous circulation, *ICU* Intensive Care Unit

**Fig. 2:**
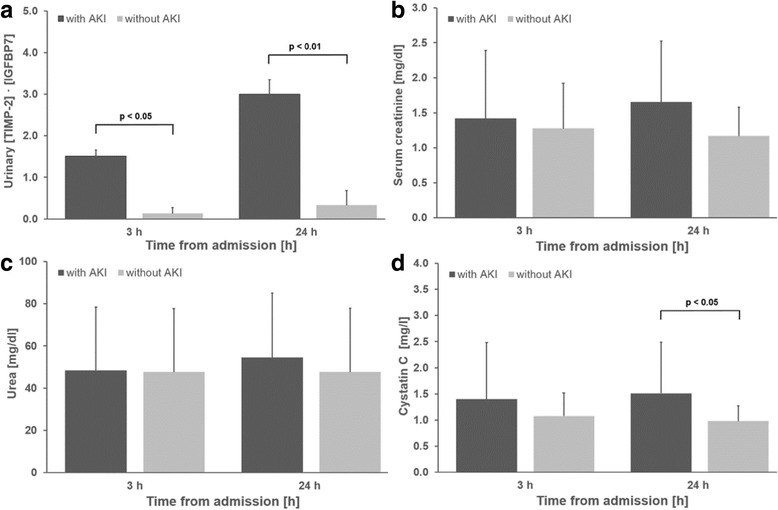
Urinary [tissue inhibitor of metalloproteinases-2]*[insulin-like growth factor-binding protein 7] ([TIMP-2]*[IGFBP7]) (**a**), serum creatinine (**b**), urea (**c**) and cystatin C concentrations (**d**) at various time points. AKI, acute kidney injury

Of the 48 study subjects, 31 (65%) developed AKI of any stage after an average of 26 ± 12 h: 10 patients (20.8%) had AKI stage 1, 11 (22.9%) had moderate AKI (stage 2) and 10 (20.8%) patients developed severe AKI (stage 3); 7 patients with AKI needed renal replacement therapy (RRT) during their hospital stay. In 19 patients AKI was diagnosed by reduced urine secretion. In the remaining 12 patients, the diagnosis of AKI was based on increases in serum creatinine: 11 patients had a previous history of advanced renal disease. Baseline serum creatinine on average was 1.40 ± 0.87 mg/dl with no difference between the AKI group and the group that did not develop AKI (1.45 ± 0.98 mg/dl vs. 1.31 ± 0.66 mg/dl, *p* = 0.61). In the AKI group, serum creatinine increased within the first 24 h (∆ + 0.24 ± 0.41 mg/dl) to a mean value of 1.69 ± 1.13 mg/dl. In contrast, patients who did not develop AKI had a decrease in serum creatinine of ∆ − 0.13 ± 0.16 to a mean value of 1.18 mg/dl ± 0.62 within the first day of admission (Fig. 2b). Urea levels remained almost constant during the observation period, whereas cystatin C levels increased in the AKI group; this increase was statistically significant after 24 h (0.98 ± 0.3 mg/l vs. 1.51 ± 0.98 mg/l, *p* < 0.05) (Fig. 2c and d).

### Fluid therapy, MAP and catecholamine regime

In both groups, fluid management was guided by continuous volumetric monitoring employing the VolumeView® system. As shown in Additional file 1: Table S1, there was no difference in the rate of fluid infusion between the two groups within the first 48 h. Patients who developed AKI required significantly greater norepinephrine doses to reach the target MAP within the first 24 h following ROSC (Additional file 1: Table S1). These patients had a continuous increase in the rate of infusion of norepinephrine and 16% of patients with AKI required additional inotropic support by epinephrine. In patients who did not develop AKI, the dose of the applied norepinephrine remained almost constant during the observation period. In contrast to the AKI group, only one patient required additional inotropic support with epinephrine. Despite lower demand for vasopressors, patients who did not develop AKI had higher MAP after 18 h of treatment (AKI group, 70 ± 10 mmHg; no AKI group, 78 ± 12 mmHg; *p* = 0.03). The required amount of dobutamine did not differ between the two groups.

### Performance of urinary [TIMP-2]·[IGFBP7] for early diagnosis of AKI

**Fig. 3:**
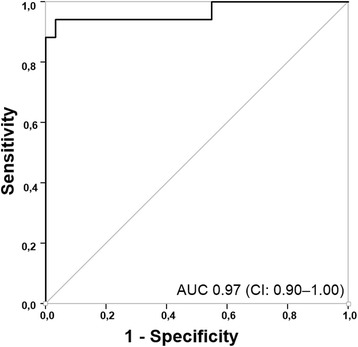
ROC curve for [tissue inhibitor of metalloproteinases-2]*[insulin-like growth factor-binding protein 7] ([TIMP-2]*[IGFBP7]) determined 3 h after out-of-hospital cardiac arrest

Patients who developed AKI had significantly higher [TIMP-2]·[IGFBP7] levels at 3 h after determination of OHCA compared to individuals that did not develop AKI (1.52 ± 0.13 vs. 0.13 ± 0.14; *p* < 0.05). As shown in Fig. 2a, the urinary [TIMP-2]·[IGFBP7] level in the AKI group peaked at a mean value of 3.00 ± 0.35 after 24 h (∆ + 1.48 ± 0.20). In contrast, urinary [TIMP-2]·[IGFBP7] levels in patients without AKI remained almost constant during the observational period. At 3 h after OHCA the area under the ROC curve (AUC) for urine [TIMP-2]*[IGFBP7] to predict AKI was 0.97 (CI 0.90–1.00) (Fig. 3). Table 2 shows the calculated sensitivities, specificities, predictive values and the Youden index at different cut-off values. The optimal [TIMP-2]·[IGFBP7] cut-off value for the prediction of AKI was 0.24. The sensitivity was 96.8% at a specificity of 94.1%. In a multivariable modelling approach the following clinical variables were included: witnessed cardiac arrest, time from determination of collapse to ROSC and dose of epinephrine during CPR. The likelihood-ratio test based on nested models with and without [TIMP-2]·[IGFBP7] (*p* = 0.031) was compared to the corresponding areas under the ROC curves of derived probability scores in a non-parametric manner (*p* = 0.049). Thus, [TIMP-2]·[IGFBP7] relevantly improved the discriminatory ability (i.e. increase in AUC from 0.86 to 0.97).

**Table 2:** Calculated sensitivities, specificities, predictive values and the Youden index at different cut-off values

Cut-off value	Sensitivity	Specificity	Youden index	PPV	NPV
0.10	100.0	52.9	52.9	79.5	100.0
0.20	96.8	88.2	85.0	93.8	93.8
0.24	96.8	94.1	90.9	96.8	94.1
0.30	71.0	94.1	65.1	95.7	64.0
0.40	67.7	94.1	61.9	95.5	61.5
0.50	54.8	94.1	49.0	94.4	53.3
0.60	45.2	94.1	39.3	93.3	48.5

*PPV* positive predictive value, *NPV* negative predictive value

### Performance of urinary [TIMP-2]·[IGFBP7] in different AKI stages

**Fig. 4:**
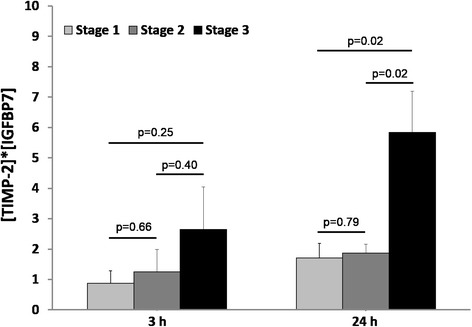
Urinary [tissue inhibitor of metalloproteinases-2]*[insulin-like growth factor-binding protein 7] ([TIMP-2]•[IGFBP7]) in different Kidney Disease Improving Global Outcomes (KDIGO) stages

When patients with AKI were stratified according to KDIGO stages, there was no significant difference in average [TIMP-2]·[IGFBP7] levels 3 h after determination of OHCA (stage 1, 0.87 ± 0.42; stage 2, 1.25 ± 0.78; stage 3, 2.62 ± 1.40; not significant). As shown in Fig. 4**,** the increase in [TIMP-2]·[IGFBP7] levels in patients reaching stage 3 was significantly greater after 24 h as compared to patients who reached stage 1–2 (*p* = 0.02).

### Prognostic accuracy of urinary [TIMP-2]•[IGFBP7] in predicting RRT

The AUC for urine [TIMP-2]*[IGFBP7] in predicting the need for RRT during the hospital stay was 0.78 (CI 0.56–0.98) for the 3-h measurement and 0.91 (0.78–1.00) for the 24-h measurement. At 3 h after determination of OHCA, sensitivity was 71.4% and specificity was 83.3% for a threshold [TIMP-2]•[IGFBP7] value of 1.0. The positive predictive value (PPV) and negative predictive value (NPV) were 55.6% and 90.9%, respectively. The calculated threshold value after 24 h was 3.67. The resulting sensitivity was 85.7% and specificity was 91.7%.The PPV was 75.0% and the NPV was 95.7%, respectively.

### Mortality and neurological outcome

Overall hospital mortality was 29%. Patients who developed AKI had greater in-hospital mortality compared to patients without AKI (42% vs. 6%; *p* < 0.01). There were 29 patients (60%) who had a favourable neurological outcome (cerebral performance categories 1 or 2) one month after OHCA. Patients who did not develop AKI had significantly better neurological outcome (no AKI group, 82% vs. AKI group, 48%; *p* = 0.03) (Table 1). According to the AUC (ROC), [TIMP-2]·[IGFBP7] carries some information to predict either neurological outcome or mortality. At the 3-h measurement the AUC was 0.73 (95% CI 0.58–0.89) to predict favourable neurological outcome and 0.82 (0.69–0.96) to predict in-hospital mortality; at the 24-h time point the AUC was 0.72 (0.56–0.87) and 0.80 (0.65–0.94), respectively.

## Discussion

In the present investigation 65% of patients with shock developed AKI on average 26 h after OHCA. We found that the novel cell cycle arrest biomarkers TIMP-2 and IGFBP7 have 94% specificity for the prediction of AKI in this high-risk population as early as 3 h after determination of OHCA. Detection of imminent AKI at this early time point could implicate measures for renal protection, such as avoidance of nephrotoxic agents or optimization of fluid management.

We further propose a new [TIMP-2]*[IGFBP7] cut-off value of 0.24 with the highest predictive value for patients under conditions that are currently considered the standard of care in patients after OHCA. The Sapphire study found that the risk of major adverse kidney events (death, dialysis or persistent renal dysfunction) within 30 days rose sharply with [TIMP-2]·[IGFBP7] > 0.3 [11]. The validation of the pre-specified cut-off values was first performed in a heterogeneous patient group not undergoing TTM [12, 19]. Later, Beitland et al. reported an incidence of AKI of 45% in patients treated with TTM after OHCA and described a urinary [TIMP-2]·[IGFBP7] level ≥ 0.36 as an independent risk marker for AKI. However, in this study urine samples were collected in a large time frame between 0 and 6 h following admission and on day 3 after OHCA; body temperature at the time of measurement was not documented [9]. Since secretion and metabolism are time and temperature dependent, we could determine the high predictive value of [TIMP-2]·[IGFBP7] at the lower cut-off value of 0.24 under precisely defined conditions 3 h after determination of OHCA at 34 °C.

In our study population pre-existing conditions did not affect the development of AKI. However, AKI was observed more frequently in patients with longer time from determination of collapse to ROSC and correlated with the total amount of adrenaline used during resuscitation. These findings are in line with previous reports which showed that the interval between collapse and ROSC and also the amount of epinephrine during CPR had an impact on the subsequent development of AKI [1, 5, 20].

In the present study the incidence of AKI was appreciably higher than in previous CA studies [7–9]. More than two thirds of patients with OHCA (65%) developed AKI. Nearly 90% of the enrolled patients had coronary heart disease. As expected, myocardial infarction was the most common cause of CA. There is good evidence that renal and cardiac function interact in critically ill patients. Other studies have successfully demonstrated that coronary artery disease and chronic heart failure are both independent risk factors for AKI after CA [7, 8]. Tujjar et al. reported a 43% incidence of AKI in patients with CA [5]. The presence of shock was thereby an independent predictor for the development of AKI. On the same lines, a recent meta-analysis of eight studies with nearly 1700 patients found that the presence of shock after resuscitation was the most consistent independent predictor of AKI [6]. All patients included in our investigation were admitted to the ICU in shock with the typical clinical signs of end-organ hypoperfusion and the need of vasopressors for circulatory support. The initial treatment of patients with shock focuses on rapid haemodynamic stabilisation with vasopressors and inotropes and volume replacement to ensure adequate organ perfusion. Norepinephrine is the vasopressor of choice to ensure haemodynamic stability in patients with cardiogenic or septic shock [2, 17]. We identified lower MAP within the first 18 h of treatment in patients with AKI. As expected, these patients required greater norepinephrine doses to reach target MAP. This finding is in line with several studies correlating vasopressor doses and hypotensive episodes with progression of AKI [5, 21].

In addition, volume therapy could have a decisive impact on the development of AKI. Hypovolaemia is an important risk factor for AKI and must be identified and treated early to preserve renal function. Nevertheless, fluid overload should be avoided because there is growing evidence that a positive fluid balance is associated with worse outcomes. Teixeira et al. analysed data on 601 patients admitted to 10 intensive care units in Italy. This study showed that a mean fluid balance > 1,3 L/day, calculated as the difference between fluid intake and fluid output, was associated with increased 28-day mortality in patients with AKI [22]. Tujjar and co-workers also found an association between high fluid balance at 48 h and AKI in patients with cardiac arrest [5]. In daily clinical practice, the assessment of the current volume status in critically ill patients represents a challenge. In contrast to traditional volume parameters like central venous pressure (CVP), haemodynamic parameters derived from transpulmonary thermodilution like global end-diastolic volume index (GEDI) or extravascular lung water (ELWI) have proven to be more reliable in the assessment of the volume status of a patient [10, 23–25]. Therefore, individual fluid management guided by means of invasive haemodynamic monitoring is part of our treatment algorithm for patients presenting with shock. However, we found no correlation between the administered amount of fluid and the incidence of AKI within the first 48 h after CA.

The KDIGO guidelines for AKI recommend different measures like volume management, haemodynamic stabilisation and avoidance or adaptation of nephrotoxic medications to prevent the development or reduce the impact of AKI [14]. Early identification of AKI could allow for more expedited induction of therapeutic measures. Meersch and co-workers examined the relationship between the measurement of [TIMP-2]·[IGFBP7] in combination with implementation of a “KDIGO bundle” in patients undergoing cardiac surgery. In this study, high-risk patients defined as having urinary [TIMP-2]·[IGFBP7] > 0.3 after cardiopulmonary bypass disconnection received a special therapy bundle in accordance with the KDIGO guidelines. These therapeutic measures reduced the frequency and severity of AKI in patients undergoing cardiac surgery, compared with standard care (55% vs. 72%; *p* = 0.004) [26]. It is currently unclear whether - and if so, to what extent - these study results are transferable to our patient collective. Our study proves the diagnostic utility of urinary [TIMP-2]·[IGFBP7] in a diverse patient population after a defined initial event of kidney injury and standardized ICU management. Urinary [TIMP-2]·[IGFBP7] identifies patients at risk of AKI as early as 3 h after OHCA. These patients need to be included in randomized clinical studies to evaluate targeted therapeutic strategies and interventions.

## Limitations

In comatose survivors of OHCA it can be difficult to assess previous renal function, thus it may not have been possible to reliably distinguish between AKI and AKI in CKD in each case. Time to ROSC and also time to urine sampling was calculated from determination of collapse to ROSC. Therefore, in patients presenting with unwitnessed arrest the exact time between collapse and ROSC may be imprecise. Our study represents the results from a prospective single-centre trial with a small sample size. This could possibly explain in part why the incidence of AKI was appreciably higher than in other CA studies. In addition, AKI was consistently classified according to the KDIGO guidelines. Other studies have used different definitions of AKI [27] or assessed only the first days of the hospital stay [9]. Another limitation may be the heterogeneity of the study population, which comprised patients in cardiogenic as well as septic shock. Therefore, our results require confirmation in a larger patient population. Finally, a previously published study protocol is not available.

## Conclusion

The presented data clearly show that measurement of [TIMP-2]•[IGFBP7] can be used to identify patients at high risk of AKI among patients with shock after OHCA. These biomarkers have good diagnostic performance for the early recognition of AKI as early as 3 h after determination of OHCA. Under conditions that are currently considered as standard care in patients who have sustained OHCA, [TIMP-2]•[IGFBP7] accurately predicts AKI with a cut-off of 0.24.

## Additional file

Additional file 1: **Table S1.** Hemodynamic parameters and cumulative amount of administered norepinephrine and fluid over the observational period of the initial 48 h upon hospital admission. MAP, mean arterial pressure; GEDI, global end diastolic volume index; ELWI, extravascular lung water index. (DOC 36 kb)

## Abbreviations

AKI: Acute kidney injury
AUC: Area under the curve
CA: Cardiac arrest
CKD: Chronic kidney disease
CPC: Cerebral performance categories
CPR: Cardiopulmonary resuscitation
CTA: Computer tomography angiography
CVP: Central venous pressure
ECMO: Extracorporeal membrane oxygenation
ELWI: Extravascular lung water index
GCS: Glasgow Coma Score
GEDI: Global end diastolic volume index
ICU: Intensive Care Unit
IGFBP7: Insulin-like growth factor-binding protein 7
KDIGO: Kidney Disease Improving Global Outcomes
MAP: Mean arterial pressure
NPV: Negative predictive value
OHCA: Out-of-hospital cardiac arrest
PCI: Percutaneous coronary intervention
PPV: Positive predictive value
RASS: Richmond agitation and sedation
ROSC: Return of spontaneous circulation
RRT: Renal replacement therapy
TIMP-2: Tissue inhibitor of metalloproteinases-2
TTM: Target temperature management
