# Supplementary material for: TIMP-2/IGFBP7 predicts acute kidney injury in out-of-hospital cardiac arrest survivors
Source: Crit Care. 2018 May 12;22:126. doi: 10.1186/s13054-018-2042-9 (PMC5948851; doi:10.1186/s13054-018-2042-9)
Supplement: Supplementary file 1 — Table S1. Hemodynamic parameters and cumulative amount of administered norepinephrine and fluid over the observational period of the initial 48 h upon hospital admission. MAP, mean arterial pressure; GEDI, global end diastolic volume index; ELWI, extravascular lung water index. (DOC 36 kb) [file 13054_2018_2042_MOESM1_ESM.doc]

| Table S1. Mean arterial pressure and cumulative amount of administered norepinephrine and fluid | | | | | | | | | | |
| --- | --- | --- | --- | --- | --- | --- | --- | --- | --- | --- |
|  | | | | | | | | | | |
|  |  | **Admission**  **to ICU** | **6 Hrs.** | **12 Hrs.** | **18 Hrs.** | **24 Hrs.** | **30 Hrs.** | **36 Hrs.** | **42 Hrs.** | **48 Hrs.** |
| MAP [mmHg] | AKI  No AKI  p-value | 78 (± 20)  74 (± 15)  0.52 | 72 (± 14)  72 (± 15)  0.99 | 69 (± 11)  74 (± 11)  0.21 | 70 (± 10)  78 (± 12)  0.03 | 64 (± 16)  73 (± 5)  0.04 | 66 (± 12)  68 (± 18)  0.79 | 69 (± 9)  74 (± 8)  0.15 | 72 (± 13)  72 (± 9)  0.93 | 72 (± 10)  72 (± 8)  0.84 |
| Norepinephrine [μg/kg/min] | AKI  No AKI  p-value | 0.16 (± 0.13)  0.08 (± 0.11)  0.03 | 0.22 (± 0.26)  0.05 (± 0.05)  0.02 | 0.28 (± 0.38)  0.08 (± 0.07)  0.06 | 0.28 (± 0.37)  0.07 (± 0.06)  0.03 | 0.29 (± 0.42)  0.07 (± 0.06)  0.04 | 0.27 (± 0.42)  0.08 (± 0.07)  0.08 | 0.30 (± 0.57)  0.08 (± 0.07)  0.14 | 0.31 (± 0.57)  0.07 (± 0.08)  0.11 | 0.32 (± 0.60)  0.08 (± 0.11)  0.12 |
| Fluid therapy [ml] | AKI  No AKI  p-value |  | 1809 (±1500)  1955 (±1348)  0.74 | 3410 (±2484)  3265 (±1603)  0.83 | 4592 (±2967)  4514 (±1860)  0.92 | 5886 (±3337)  5849 (±2228)  0.97 | 7401 (±4087)  6984 (±2683)  0.71 | 8367 (±4442)  8093 (±3016)  0.82 | 8117 (±4861)  9140 (±3125)  0.99 | 9923 (±5169)  9711 (±2504)  0.87 |
